# Bone Marrow Necrosis After Curative Allogeneic Hematopoietic Stem Cell Transplant Associated with Deep Diving

**DOI:** 10.5146/tjpath.2024.13488

**Published:** 2026-05-30

**Authors:** Hajdhica Thanasi, Daniele Avenoso, Liron Barnea Slonim

**Affiliations:** Department of Histopathology, King’s College Hospital NHS Foundation Trust, LONDON, UNITED KINGDOM;Department of Hemato-Oncology, University of Milan, Specialty School of Pathological Anatomy, MILAN, ITALY; Department of Haematological Medicine, King's College Hospital NHS Foundation Trust, LONDON, UNITED KINGDOM; Department of Histopathology, King’s College Hospital NHS Foundation Trust, LONDON, UNITED KINGDOM

**Keywords:** Bone marrow necrosis, Bone marrow transplant, Deep diving

## Abstract

Bone marrow necrosis, albeit an infrequent finding, is usually associated with highly proliferative malignant disorders, such as leukemia, lymphoma, or sickle cell anemia, solid tumor metastasis, and infections. It is also a typical finding in Caisson disease and in divers that developed decompression illness. Herein we report a case of bone marrow necrosis post deep diving in a bone marrow transplant recipient.

## Introduction

Allogeneic hematopoietic stem cell transplantation (allo-HSCT) is a crucial therapeutic intervention for patients with aggressive myeloid malignancies (1) and myelodysplastic/myeloproliferative neoplasm with neutrophilia (MDS/MPN with neutrophilia), formerly known as atypical chronic myeloid leukemia (aCML)(2). This aggressive malignancy, characterized by the absence of the BCR-ABL1 fusion gene, has limited treatment options, and allo-HSCT offers a potential cure by replacing the patient's diseased hematopoietic system with healthy donor cells. Clinical studies indicate that allo-HSCT can lead to long-term remission in MDS/MPN with neutrophilia patients (3,4). Careful histological evaluation of bone marrow post-transplant is essential to monitor engraftment and detect potential complications such as GVHD and disease relapse. This evaluation involves regular bone marrow biopsies and assessments of cellular morphology and chimerism status to ensure the transplanted cells are proliferating as expected. Close monitoring allows for early intervention in case of impending disease relapse or mixed chimerism.

## Case presentation

A 63-year-old man with a background of MDS/MPN with neutrophilia underwent curative allo-HSCT in May 2022. The post-transplant period was uneventful and he attended the transplant clinic on regular basis. The disease evaluations performed after one, three, and six months were in keeping with complete remission (CR); indeed the patient was able to return to his normal life activities, including deep diving in Malta.

Before the disease re-evaluation one year after allo-HSCT, he spent two weeks in Malta where he performed dives up to 25 meters deep.

The clinical evaluation was unremarkable with full blood count parameters within the range of normality.

**Figure 1:**
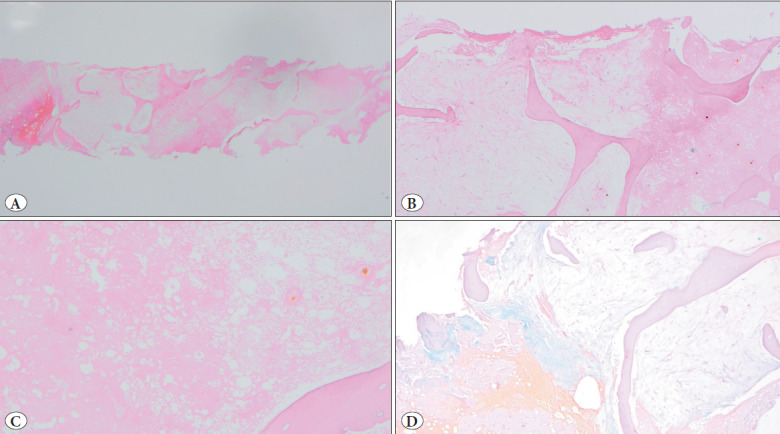
A) Low magnification of necrotic bone marrow (10x). B,C) Higher magnification of bone marrow biopsy showing mostly necrotic bone marrow without hematopoiesis, (20x, 40x). D) Alcian blue highlights a small area of serous atrophy (20x).

The bone marrow biopsy showed mostly necrotic bone and bone marrow with and focal acellular marrow with serous atrophy Figure 1. No hematopoietic cells were seen. Immuno-histochemistry showed negative CD34, CD117, E-cadherin, MPO, and CD61; Alcian blue highlighted a small area of serous atrophy Figure 1.

## Discussion

Dysbaric osteonecrosis is a typical finding in Caisson disease (5) and in divers who develop decompression illness (6). This condition arises due to the formation of nitrogen bubbles in the blood and tissues when a diver ascends too quickly, causing damage to bone and other tissues. The patchy involvement of gas necrosis can explain a bone marrow biopsy in keeping with CR when it was performed on the opposite iliac crest side. In this case, the patient's diving history likely contributed to the observed bone marrow necrosis. Notably, the patient is a regular diver and had a previous history of bilateral avascular hip necrosis prior to the onset of MDS/MPN with neutrophilia. This background emphasizes the importance of considering non-hematologic factors when evaluating bone marrow findings. Bone marrow necrosis, albeit an infrequent finding, is usually associated with highly proliferative malignant disorders, such as leukaemia, lymphoma, or sickle cell anemia, solid tumor metastasis, and infections. Its occurrence post-allo-HSCT can be particularly concerning, as it may mimic disease relapse or other serious complications. In this patient, however, the necrosis appears to be linked to external factors rather than the underlying malignancy or transplant-related complications. The detailed histological examination and immunohistochemical staining were crucial in ruling out hematopoietic cell involvement and confirming the necrotic nature of the marrow.

This case illustrates the need for careful review of clinical history, including recreational activities, when interpreting post-transplant biopsy results. Divers, especially those with a history of dysbaric conditions, present unique diagnostic challenges. The finding of bone marrow necrosis in this patient underscores the complexity of post-transplant care and the need for interdisciplinary communication.

The importance of intertalk between the hematopathologist and the clinician cannot be overstated. Detailed clinical information, including the patient's activities and medical history, is essential for accurate diagnosis and appropriate management. In this case, the absence of hematopoietic cells and the presence of necrotic bone with serous atrophy suggested a non-malignant aetiology, which was corroborated by the patient's diving history and prior avascular necrosis.

This case also highlights the broader implications of allo-HSCT in patients with MDS/MPN with neutrophilia. While the procedure offers a potential cure, the long-term success and quality of life post-transplant can be influenced by a range of factors beyond the immediate transplant-related risks. Regular follow-up and comprehensive assessments, incorporating both hematologic and non-hematologic parameters, are essential for optimizing patient outcomes.

In conclusion, this case emphasizes the multifaceted nature of post-allo-HSCT care. The occurrence of bone marrow necrosis in a patient with a history of deep diving activities underscores the need for a holistic approach to patient management. Interdisciplinary collaboration and thorough clinical evaluation are critical for distinguishing between transplant-related complications and external factors, ultimately guiding appropriate treatment strategies and ensuring the best possible outcomes for patients undergoing allo-HSCT.

## Conflict of Interest

The authors declare that they have no conflict of interest for this article.
